# Absent/weak CD44 intensity and positive human papillomavirus (HPV) status in oropharyngeal squamous cell carcinoma indicates a very high survival

**DOI:** 10.1002/cam4.90

**Published:** 2013-06-14

**Authors:** Anders Näsman, Cecilia Nordfors, Nathalie Grün, Eva Munck-Wikland, Torbjörn Ramqvist, Linda Marklund, David Lindquist, Tina Dalianis

**Affiliations:** 1 Department of Oncology-Pathology, Karolinska InstitutetStockholm, Sweden; 2 Department of Oto-Rhino-LaryngologyHead and Neck SurgeryKarolinska Institutet, Karolinska University HospitalStockholm, Sweden; 3 Department of Oncology, Umeå UniversityUmeå, Sweden

**Keywords:** CD44, human papillomavirus, oropharyngeal cancer, prognosis

## Abstract

Patients with human papillomavirus DNA positive (HPV_DNA_+) oropharyngeal squamous cell carcinoma (OSCC) have better clinical outcome than those with HPV DNA negative (HPV_DNA_−) OSCC upon intensive oncological treatment. All HPV_DNA_+ OSCC patients may not require intensive treatment, however, but before potentially deintensifying treatment, additional predictive markers are needed. Here, we examined HPV, p16^INK4a^, and CD44 in OSCC in correlation to clinical outcome. Pretreatment tumors from 290 OSCC patients, the majority not receiving chemotherapy, were analyzed for HPV DNA by Luminex and for p16^INK4a^ and CD44 by immunohistochemistry. 225/290 (78%) tumors were HPV_DNA_+ and 211/290 (73%) overexpressed p16^INK4a^, which correlated to presence of HPV (*P* < 0.0001). Presence of HPV DNA, absent/weak CD44 intensity staining correlated to favorable 3-year disease-free survival (DFS) and overall survival (OS) by univariate and multivariate analysis, and likewise for p16^INK4a^ by univariate analysis. Upon stratification for HPV, HPV_DNA_+ OSCC with absent/weak CD44 intensity presented the significantly best 3-year DFS and OS, with >95% 3-year DFS and OS. Furthermore, in HPV_DNA_+ OSCC, p16^INK4a^+ overexpression correlated to a favorable 3-year OS. In conclusion, patients with HPV_DNA_+ and absent/weak CD44 intensity OSCC presented the best survival and this marker combination could possibly be used for selecting patients for tailored deintensified treatment in prospective clinical trials.

Absence of/weak CD44 or presence of human papillomavirus (HPV) DNA was shown as a favorable prognostic factors in tonsillar and tongue base cancer. Moreover, patients with the combination of absence of/weak CD44 and presence of HPV DNA presented a very favorable outcome. Therefore, we suggest that this marker combination could potentially be used to single out patients with a high survival that could benefit from a de-escalated oncological treatment.

## Introduction

Recent reports from several countries indicate an increased incidence of oropharyngeal squamous cell carcinoma (OSCC) 1–5, where tonsillar squamous cell carcinoma (TSCC) and base of tongue squamous cell carcinoma (BOTSCC) dominate. This increase has mainly been attributed to human papillomavirus (HPV) infection 2.

Furthermore, patients with HPV DNA positive (HPV_DNA_+) OSCC have been reported to have a better 5-year overall survival (OS) compared with those with HPV_DNA_− OSCC (80% and 40%, respectively), the latter similar to that of other head–neck squamous cell carcinoma (HNSCC) patients 5,6.

Lately, as a consequence of the low survival in HNSCC, oncologic treatment has been intensified with chemo-radiotherapy and epidermal growth factor receptor (EGFR) inhibitors 7. Many patients with HPV_DNA_+ OSCC may not benefit from this intensified treatment, and could potentially be cured by radiotherapy (RT) alone, with possibly less severe sequele. To better identify patients with a favorable prognosis, before potentially reducing treatment, additional predictive markers are needed 5.

Expression of CD44, a cell adhesion glycoprotein participating in epithelial cell–stroma interactions and important for tumor invasion and metastasis 8, has previously been described as a prognostic marker in many cancers 9–10. Moreover, high CD44 expression has been correlated to worse prognosis in HNSCC 11–14, and in a pilot study from the rural county of Dalarna, Sweden, we found that medium/strong CD44 intensity staining was a negative prognostic factor in tonsillar and base of tongue cancer 11. However, we could not correlate this finding to HPV status due to limited numbers of patients.

Furthermore, in the present literature there are different standard procedures for defining HPV status. HPV status can, for example, be defined as presence of HPV DNA alone, or HPV DNA together with overexpression of p16^INK4a^. However, overexpression of p16^INK4a^ alone has also been used as a surrogate marker of functionally active HPV 12–13.

Here, in this larger Stockholm, Sweden cohort of HPV+ and HPV− OSCC, where 80% of the patients did not receive chemotherapy, we have evaluated CD44 intensity staining and p16^INK4a^ in relationship to HPV status and in relation to OS and DFS.

## Patients, Materials, and Methods

### Patients

2000–2007, 385 patients were diagnosed with TSCC (ICD-10 C09.0-9) and BOTSCC (ICD-10 C01.9) in the county of Stockholm, and of these 290 with available pretreatment biopsies and treated with intention to cure were included in the study. For most patients (*n* = 229, 79%) treatment consisted of conventional RT (2.0 Gy/day, for 6.5–7 weeks, total dose: 68 Gy) or accelerated RT (1.1 + 2.0 Gy/day for 4.5 weeks, total dose: 68 Gy), while a minority (*n* = 61, 21%) also had induction chemotherapy followed by concomitant RT. Furthermore, some patients also received interstitial radiation (brachytherapy) (total dose of 78 Gy). Finally, patients with nodal disease also underwent neck dissection, 6–8 weeks after completed RT. Patients were thereafter followed up by clinical examination every 3 months the first 2 years, and every 6 months the third year.

Patients' characteristics were obtained from clinical records. The study was conducted according to ethical permissions 2005/431-31/4, 2005/1330-32, and 2009/1278-31/4 from the Regional Ethical Committee at Karolinska Institutet, Stockholm, Sweden.

### HPV DNA analysis

DNA was extracted from 30 μm paraffin-embedded tonsillar tumor biopsies as described previously 2. Blank control samples were treated in the same way to exclude cross-contamination between samples. Presence of HPV DNA and type and betaglobin was analyzed by the Luminex method 14.

### Immunohistochemistry

In brief, tumor sections (4–5 μm) were deparaffinized, rehydrated, rinsed in water, followed by antigen retrieval in citrate buffer (pH 6) for 20 min. The slides were then left for 10 min in 0.5% H_2_O_2_ in water and washed in phosphate buffered saline (PBS). Blocking was done with 1% horse serum in PBS in a moist chamber for 40 min before the sections were stained with the primary antibody (mAb CD44 [clone: DF1485, dilution 1:100, Dako, Glostrup, Denmark] and mAb p16^INKA4a^ [clone: JC8, dilution 1:100, Santa Cruz Biotech, Dallas, TX]) at +8°C over night. The avidin–biotin–peroxidase complex (ABC) kit (Vectastain, Vector Laboratories, Burlingame, CA) was used for antigen detection according to the manufacturer. Slides were developed in chromogen 3'-diaminobenzydine (DAB) (Vector Laboratories) with hematoxylin as a counter stain.

### Evaluation of immunohistochemistry staining

The fraction of CD44 positive cells was evaluated semi-quantitatively in four grades of percentages of stained malignant cells: 0 (0%), 1 (1–25%), 2 (26–75%), or 3 (76–100%) and the intensity of the staining was scored separately and evaluated as absent, weak, moderate, and strong staining 11. The fraction and intensity of p16^INK4a^ positive cells was evaluated and samples with strong p16^INK4a^ staining in >70% positive cells were considered as p16^INK4a^ positive (p16^INK4a^+) 15. All evaluations were conducted by two independent researchers (A. N. and C. N.) blinded for clinical data and outcome.

### Statistical analyses

Patient characteristics were analyzed with the Chi^2^-test and independent *T*-test when appropriate. Two-sided *P*-values were reported for all analyses.

Disease-free survival (DFS) was defined from the date of diagnosis to the date of the last known occasion that the patient was disease free, or the date of disease recurrence (local, regional, or distant recurrence). Death without documented recurrence was censored at the date of death. OS was defined as time from the date of diagnosis until the date of death of any reason. Cumulative survival was calculated and presented with the Kaplan–Meier method and analyzed with the log-rank test. Univariate and multivariate Cox proportional models were used to calculate the Hazard ratio (HR). All above described analyses were performed in SPSS (IBM SPSS Statistics, version 20, Stockholm, Sweden).

## Results

### Patients, HPV_DNA_, and p16^INK4a^ status

When this study was initiated in 2011, 290 pretreatment tumor biopsies were available from patients with TSCC and BOTSCC diagnosed between 2000 and 2007 and treated with intention to cure. The characteristics of the patients and their tumors are summarized in Table 1. Of these tumors, 78% (225/290) were HPV_DNA_+ with HPV-16 as the dominant type (*n* = 211, 94%), followed by HPV33 (*n* = 8), HPV35 (*n* = 3), HPV56 (*n* = 1), HPV58 (*n* = 1), and HPV59 (*n* = 1). Moreover, 73% (211/290) of the tumors were p16^INK4a^+, and HPV_DNA_+ tumors expressed p16^INK4a^ to a significantly higher level than HPV_DNA_− tumors (203/225, 90% and 8/65, 12%, respectively, *P* < 0.0001). Furthermore, patients with HPV_DNA_+ tumors were younger at diagnosis (mean age 59.8 years vs. 63.4 years, *P* = 0.01) and presented with a greater nodal disease (N2–N3 vs. N0–N1) (*P* = 0.01), smaller tumors (T1–T2 vs. T3–T4) (*P* = 0.01) and a higher clinical stages (III–IV vs. I–II) (*P* < 0.01). No significant differences were observed in tumor localization, sex, or presence of distant metastases between patients with HPV_DNA_+ and HPV_DNA_− tumors.

**Table:** Patient characteristics

	HPV_DNA_ positive patients (*n* = 225)^1^			HPV_DNA_ negative patients (*n* = 65)					*P*-value	All patients (*n* = 290)							
Count	%	Count	%	Count	%				
Diagnosis							
Base of tounge squamous cell carcinoma	60	27	20	31	NS^2^	80	28
Tonsillar squamous cell carcinoma	165	73	45	69		210	72
Sex							
Female	58	26	18	28	NS^2^	76	26
Male	167	74	47	72		214	74
Age (years)							
Mean age	59.8		63.4		0.01^3^	60.6	
Median age	59		62			60	
Range	30–90		44–82			30–90	
Percentiles							
25	53		56.5			54	
50	59		62			60	
75	66		70.5			67	
TNM							
T1	54	24	13	20	0.04^2^	67	23
T2	82	36	14	22		96	33
T3	45	20	17	26		62	21
T4	44	20	21	32		65	22
N0	35	16	27	42	<0.001^2^	62	21
N1	55	24	11	17		66	23
N2a	39	17	3	5		42	14
N2b	67	30	10	15		77	27
N2c	22	10	9	14		31	11
N3	7	3	5	8		12	4
M0	222	99	64	98	NS^2^	286	99
M1	2	1	1	2		3	1
MX	1	0	0	0		1	0
Stage							
I	2	1	8	12	0.001^2^	10	3
II	15	7	5	8		20	7
III	60	27	17	26		77	27
IV	148	66	35	54		183	63
CD44 expression							
Absent	20	9	2	3	0.002^2^	22	8
1–25%	28	12	2	3		30	10
25–75%	45	20	6	9		51	18
75–100%	132	59	55	85		187	64
CD44 intensity							
Absent	20	9	2	3	0.002^2^	22	8
Weak	53	24	4	6		57	20
Medium	53	24	17	26		70	24
Strong	99	44	42	65		141	49
p16^INK4a^							
Negative	22	10	57	88	<0.0001^2^	79	27
Positive	203	90	8	12		211	73

HPV_DNA_, human papillomavirus DNA.

1 HPV-16 (*n* = 211); HPV-33 (*n* = 8); HPV-35 (*n* = 3); HPV-56 (*n* = 1); HPV-58 (*n* = 1) and HPV-59 (*n* = 1).

2 Chi-square test.

3 Independent *T*-test.

### CD44 expression without or with HPV_DNA_ status and clinical parameters

CD44 expression was evaluated both by staining intensity and by the fraction of positive cells (Fig. S1). In all, 268/291 (92%) of the tumors expressed CD44, and the majority had a strong intensity staining (53%), while 26% and 21%, respectively, stained intermediately or weakly (Table 1). Patients with CD44+ tumors (defined by dichotomization at intensity >weak or by >26% positive cells), presented significantly more often with larger, higher differentiated tumors, but no significant differences were observed in tumor localization (tonsil or tongue base), stage, or age between patients with CD44+ and CD44− tumors (data not shown).

When the CD44 intensity, dichotomized as absent/weak versus medium/strong, was compared between HPV_DNA_+ and HPV_DNA_− tumors, the HPV_DNA_+ tumors expressed high intensity CD44 staining to a significantly lesser extent than HPV_DNA_− tumors (HPV_DNA_+: 152/225, 65% vs. HPV_DNA_−: 59/65, 91%, *P* < 0.001). If CD44 intensity was dichotomized as absent/weak/medium versus strong, HPV_DNA_+ OSCC still expressed CD44 to a lesser extent than HPV_DNA_− OSCC (HPV_DNA_+: 99/225, 44% vs. HPV_DNA_− 42/65, 65%, *P* = 0.005) (Table 1).

When the fraction of cells expressing CD44, dichotomized as 0–75% and >75%, was compared between HPV_DNA_+ and HPV_DNA_− tumors, the HPV_DNA_+ tumors also expressed CD44 to a significantly lesser extent than HPV_DNA_− tumors (132/225, 59% vs. 55/65, 85%, *P* < 0.0001) (Table 1). The same correlation was observed when a cut-off was applied at <25% positive cells (177/225, 79% vs. 61/65, 94%, *P* = 0.003) (Table 1).

However, if CD44 expression was grouped as absent or present, no significant difference was observed between HPV_DNA_+ and HPV_DNA_− tumors, where 205/225, 91% of the HPV+ and 63/65, 97% of the HPV_DNA_− tumors expressed CD44 (Table 1).

### Effects of HPV_DNA_, p16^INK4a^, absent/weak CD44 intensity, and age on clinical outcome

**Figure 1:**
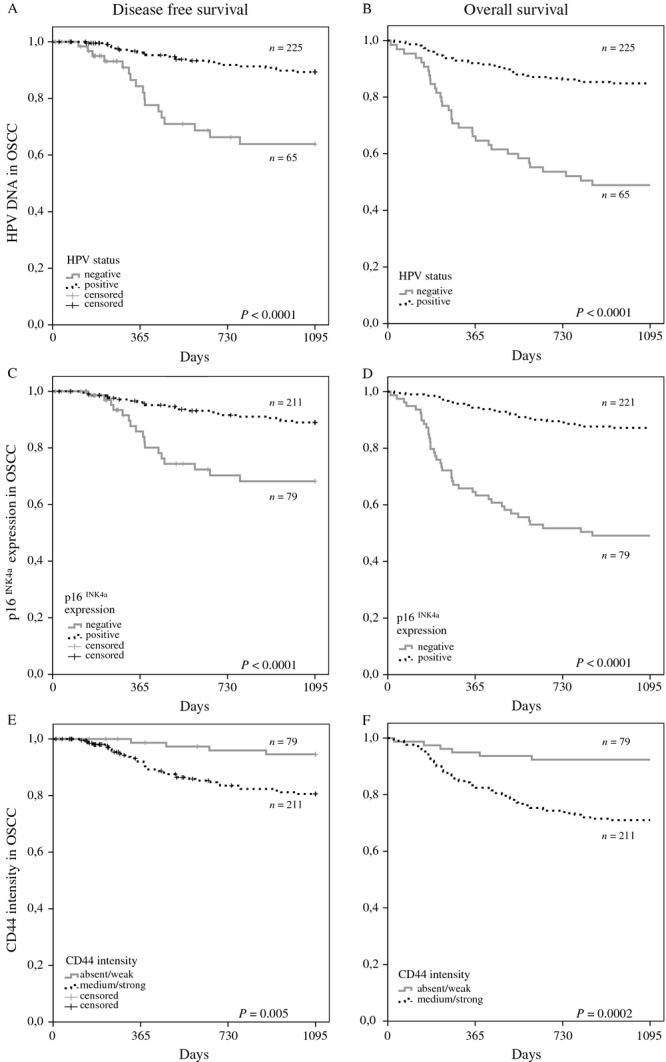
Disease-free survival (DFS) and overall survival (OS) analyzed by human papillomavirus DNA (HPV_DNA_), p16^INK4a^ overexpression and CD44 intensity staining as visualized by Kaplan–Meier diagrams. (A) DFS and (B) OS by presence and absence of HPV_DNA_; (C) DFS and (D) OS by presence and absence of p16^INK4a^ overexpression; (E) DFS and (F) OS by absent/weak and medium/strong CD44 intensity staining. *P*-values are calculated with the log-rank test. *n* denotes the number of patients in each group.

The univariate analysis showed that patients with HPV_DNA_+ tumors were less likely to relapse in disease (89% vs. 64%) (HR 0.24, 95% CI: 0.13–0.45, *P* < 0.0001) or to die of any cause (85% vs. 49%) (HR 0.23, 95% CI: 0.14–0.37, *P* < 0.0001) within 3 years, relative to patients with HPV_DNA_− tumors (Fig. 1A and B, and Table 2).

**Table:** Univariate and multivariate analyses of HPV_DNA_, CD44 intensity and clinical parameters for 3-year disease free and overall survival

	Univariate analysis							Multivariate analysis^1^												
Disease-free survival				Overall survival							Disease-free survival										Overall survival												
HR	95% CI	*P*-value	HR	95% CI	*P*-value	HR	95% CI	*P*-value	HR	95% CI	*P*-value
HPV_DNA_												
Absent	1	(ref)		1	(ref)		1	(ref)		1	(ref)	
Present	0.24	0.13–0.45	<0.0001	0.23	0.14–0.37	<0.0001	0.31	0.16–0.62	0.001	0.27	0.16–0.44	<0.0001
CD44_intensity_												
Absent/weak	1	(ref)		1	(ref)		1	(ref)		1	(ref)	
Medium/strong	4.0	1.4–11.1	0.009	4.3	1.8–9.9	0.001	3.0	1.1–8.7	0.046	3.1	1.3–7.4	0.010
Age												
>67 years	1	(ref)		1	(ref)		1	(ref)		1	(ref)	
60–67 years	1.0	0.48–2.2	0.94	0.60	0.34–1.1	0.09	0.73	0.41–1.3	0.31	0.75	0.42–1.4	0.35
54–60 years	0.56	0.23–1.4	0.20	0.39	0.19–0.78	0.007	0.42	0.21–0.85	0.016	0.44	0.22–0.88	0.020
<54 years	0.19	0.054–0.67	0.009	0.29	10.14–0.61	0.001	0.42	0.20–0.92	0.029	0.42	0.20–0.90	0.027
Stage												
I–II	1	(ref)		1	(ref)		1	(ref)		1		
III–IV	0.62	0.26–1.5	0.28	1.4	0.57–3.6	0.44	1.0	0.41–2.5	0.98	2.4	0.95–6.1	0.065
Sex												
Female	1	(ref)		1	(ref)		1	(ref)		1	(ref)	
Male	1.2	0.58–2.3	0.68	0.7	0.39–1.3	0.24	0.99	0.50–2.0	0.97	0.59	0.34–1.1	0.086
Tumour site												
Tonsillar SCC	1	(ref)		1	(ref)		1	(ref)		1	(ref)	
Base of tongue SCC	1.1	0.53–2.2	0.81	0.95	0.56–1.6	0.85	1.3	0.63–2.7	0.48	1.0	0.59–1.7	0.96

HPV_DNA_, human papillomavirus DNA; HR, hazard ratio; CI, confidence interval; SCC, squamous cell carcinoma.

1 All listed factors are included in the regression model.

Similarly, patients with p16^INK4a^+ tumors had also a favorable 3-year DFS (89% vs. 68%) and OS (87% vs. 49%) (HR 0.30, 95% CI: 0.16–0.57, *P* = 0.0001 and HR 0.18, 95% CI: 0.11–0.30, *P* < 0.0001, respectively) (data not shown).

Moreover, when CD44 intensity staining was evaluated as previously described 11, OSCC with absent/weak CD44 staining intensity had a significantly better 3-year DFS (95% vs 81%) and OS (92% vs. 71%) (HR 4.0, 95% CI: 1.4–11.1, *P* = 0.001 and HR 4.3, 95% CI: 1.8–9.9, *P* = 0.001, respectively) compared to OSCC with medium/strong CD44 staining intensity (Fig. 1C and D, and Table 2).

Additionally, age was divided in percentiles and the effect on outcome was analyzed. A lower age was significantly correlated to a better DFS and OS in the univariate analysis (Table 2).

A multivariate including HPV status, CD44 intensity staining, age, sex, stage, and tumor site was performed, but here p16^INK4a^ was excluded due to the high correlation between HPV_DNA_ and p16^INK4a^ overexpression. Patients with HPV_DNA_+ tumors had a favorable DFS and OS (HR 0.31, 95% CI: 0.16–0.62, *P* = 0.001 and 0.27, 95% CI: 0.16–0.44, *P* < 0.001, respectively) (Table 2). Similar trends in DFS and OS were observed for patients with tumors expressing CD44 when assaying for staining intensity (CD44 absent/weak intensity vs. medium/strong) (HR 3.0, 95% CI: 1.1–8.7, *P* = 0.046 and HR 3.1, 95% CI: 1.3–7.4, *P* = 0.010, respectively) (Table 2). Finally, a lower age was also significantly correlated to a better DFS and OS in the multivariate analysis.

### Effects on survival combining HPV_DNA_/CD44 or HPV_DNA_/p16INK4a, as well as HPV_DNA_/age

**Figure 2:**
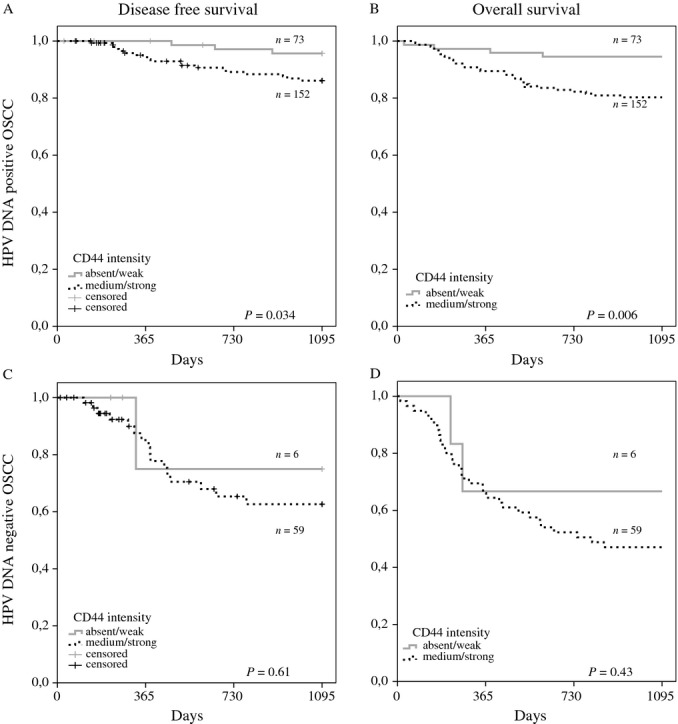
Disease-free survival (DFS) and overall survival (OS) analyzed by CD44 intensity staining and p16^INK4a^ overexpression stratified for HPV_DNA_ status, as visualized by Kaplan–Meier diagrams. (A) DFS and (B) OS by absent/weak and medium/strong CD44 intensity staining, respectively, in HPV_DNA_+ OSCC. (C) DFS and (D) OS by absent/weak and medium/strong CD44 intensity staining, respectively, in HPV_DNA_− OSCC. HPV_DNA_, human papillomavirus DNA; OSCC, oropharyngeal squamous cell carcinoma.

Strong evidence suggests that “HPV positive” OSCC and “HPV negative” OSCC are different disease entities with different characteristics and should be separated when analyzed. Hence, the cohort was divided into an HPV_DNA_+ and HPV_DNA_− cohort and the influence of CD44 and p16^INK4a^ as prognostic factors was also analyzed (Fig. 2, and Table 3A and B).

**Table:** Univariate and multivariate analyses in (A) HPV_DNA_+ and (B) HPV_DNA_− patients of CD44_intensity_, p16^INK4a^ expression and clinical parameters for 3-year disease free and overall survival

	Univariate analysis							Multivariate analysis^1^												
Disease-free survival				Overall survival							Disease-free survival										Overall survival												
HR	95% CI	*P*-value	HR	95% CI	*P*-value	HR	95% CI	*P*-value	HR	95% CI	*P*-value
(A) HPV_DNA_+												
CD44_intensity_												
Absent/weak	1	(ref)		1	(ref)		1	(ref)			(ref)	
Medium/strong	3.4	1.0–11.6	0.047	3.9	1.4–10.9	0.011	3.7	1.1–12.6	0.036	3.4	1.2–9.8	0.024
p16^INK4a^ expression												
Absent	1	(ref)		1	(ref)		1	(ref)		1	(ref)	
Present	0.67	0.16–2.9	0.59	0.16	0.076–0.32	<0.0001	0.42	0.088–2.0	0.28	0.089	0.039–0.20	<0.0001
Age												
>67 years	1	(ref)		1	(ref)		1	(ref)		1	(ref)	
60–67 years	0.83	0.33–2.1	0.69	0.40	0.17–0.93	0.033	0.82	0.32–2.1	0.68	0.37	0.16–0.87	0.023
54–60 years	0.18	0.040–0.85	0.031	0.22	0.073–0.64	0.006	0.15	0.031–0.71	0.017	0.15	0.047–0.45	0.001
<54 years	0.16	0.034–0.72	0.024	0.23	0.084–0.62	0.004	0.12	0.025–0.61	0.010	0.13	0.043–0.37	<0.0001
Stage												
I–II	1	(ref)		1	(ref)		1	(ref)		1		
III–IV	0.74	0.17–3.2	0.69	0.79	0.24–2.6	0.70	0.76	0.17–3.3	0.71	0.64	0.19–2.2	0.48
Sex												
Female	1	(ref)		1	(ref)		1	(ref)		1	(ref)	
Male	1.3	0.54–3.2	0.55	0.88	0.40–1.9	0.74	1.2	0.47–3.1	0.70	0.53	0.23–1.2	0.13
Tumour site												
Tonsillar SCC	1	(ref)		1	(ref)		1	(ref)		1	(ref)	
Base of tongue SCC	1.2	0.44–3.2	0.74	0.84	0.4–1.8	0.64	1.4	0.51–3.9	0.52	0.95	0.045–2.0	0.90
(B) HPV_DNA_−												
CD44_intensity_												
Absent/weak	1	(ref)		1	(ref)		1	(ref)		1	(ref)	
Medium/strong	1.7	0.22–12.6	0.62	1.8	0.43–7.4	0.43	1.9	0.24–15.3	0.55	2.4	0.25–11.1	0.26
p16^INK4a^ expression												
Absent	1	(ref)		1	(ref)		1	(ref)		1	(ref)	
Present	0.85	0.19–3.7	0.83	0.84	0.30–2.4	0.75	1.4	0.27–7.4	0.69	0.66	0.21–2.0	0.46
Age												
>67 years	1	(ref)		1	(ref)		1	(ref)		1	(ref)	
60–67 years	1.7	0.48–6.0	0.41	1.0	0.44–2.4	0.95	1.9	0.50–7.5	0.34	1.5	0.62–3.7	0.37
54–60 years	1.9	0.55–6.9	0.31	0.78	0.30–2.0	0.60	2.4	0.67–9.0	0.18	1.0	0.39–2.8	0.94
<54 years	0.44	0.049–3.9	0.46	0.69	0.22–2.2	0.53	0.43	0.041–4.5	0.48	1.1	0.32–3.7	0.89
Stage												
I–II	1	(ref)		1	(ref)		1	(ref)		1		
III–IV	1.2	0.4–3.8	0.72	5.1	1.2–21.5	0.025	1.3	0.40–4.4	0.64	6.3	1.5–27.0	0.014
Sex												
Female	1	(ref)		1	(ref)		1	(ref)		1	(ref)	
Male	0.71	0.25–2.0	0.52	0.44	0.18–1.1	0.07	0.60	0.20–1.8	0.36	0.39	0.16–0.97	0.043
Tumour site												
Tonsillar SCC	1	(ref)		1	(ref)		1	(ref)		1	(ref)	
Base of tongue SCC	1.3	0.46–3.7	0.62	1.4	0.64–3.0	0.42	1.5	0.50–4.5	0.46	1.2	0.56–2.7	0.60

HPV_DNA_, human papillomavirus DNA; HR, hazard ratio; CI, confidence interval; SCC, squamous cell carcinoma.

1 All listed factors are included in the regression model.

As shown in Figure 2A and B, patients with HPV_DNA_+ tumors and an absent/weak CD44 intensity had a significantly better DFS (96% vs. 86%) and OS (95% vs. 80%) compared with patients with HPV_DNA_+ and medium/strong CD44 intensity tumor expression (*P* = 0.034 and *P* = 0.060, respectively). This was the case irrespective if the patients were treated with RT alone or induction chemotherapy followed by RT (data not shown). In the HPV_DNA_− cohort absent/weak CD44 as compared to medium/strong CD44 intensity staining showed a similar tendency with 75% versus 63% DFS and 67% versus 47% OS, respectively, but these differences were not statistically significant (Fig. 2C and D).

Similarly, when analyzed separately for HPV_DNA_+ and HPV_DNA_− tumors, in the multivariate analyses adjusted for sex, stage, age, and tumor site, CD44 expression was still correlated to a favorable DFS and OS for the HPV_DNA_+ group, but not the HPV_DNA_− group (Table 3A and B).

Only a minority of patients with HPV_DNA_+ tumors were p16^INK4a^ negative and vice versa. When DFS and OS were analyzed in the HPV_DNA_+ and HPV_DNA_− groups, a statistically significant difference was observed only in OS between patients with HPV_DNA_+/p16^INK4a^+ and patients with HPV_DNA_+/p16^INK4a^− tumors (univariate DFS: 0.59 and OS: *P* < 0.0001, respectively) (Table 3A). Likewise, when p16^INK4a^ overexpression was analyzed separately for HPV_DNA_+ and HPV_DNA_− tumors in the multivariate analyses adjusted for sex, stage, age, and tumor site, there was only a significant correlation to OS in the HPV_DNA_+ group and not in the HPV_DNA_− group (multivariate DFS: *P* = 0.28 and OS: *P* < 0.0001, respectively) (Table 3A and B).

Finally, a lower age upon diagnosis correlated significantly to a more favorable DFS and OS in the HPV_DNA_+ group both in the univariate and the multivariate analysis (Table 3A). A similar tendency was observed in the HPV_DNA_− patient group, but did not reach statistical significance (Table 3B).

### Effects on survival combining HPV_DNA_ with p16^INK4a^ expression and CD44 expression

**Figure 3:**
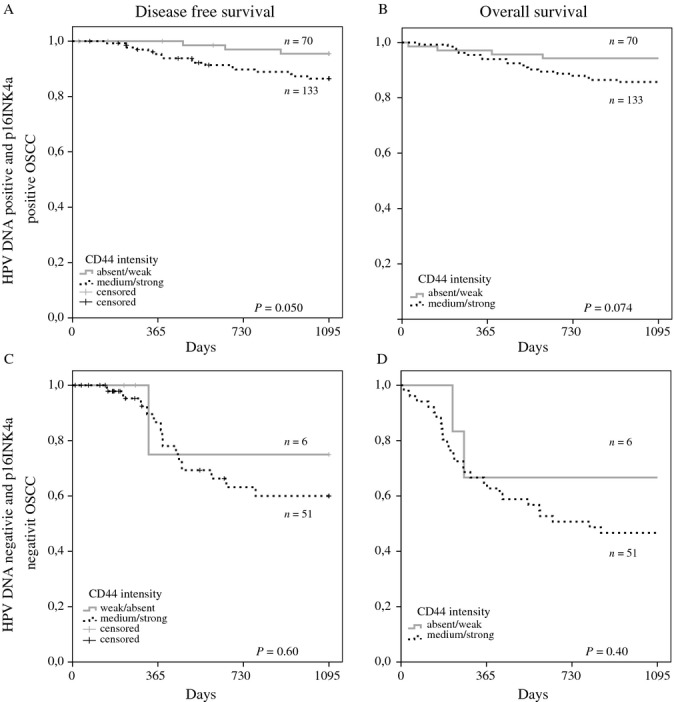
Disease-free survival (DFS) and overall survival (OS) analyzed by CD44 intensity staining, stratified for HPV_DNA_ status and p16^INK4a^ overexpression, as visualized by Kaplan–Meier diagrams. (A) DFS and (B) OS by absent/weak and medium/strong CD44 intensity staining, respectively, in HPV_DNA_+/p16^INK4a^+ OSCC. (C) DFS and (D) OS by absent/weak and medium/strong CD44 intensity staining, respectively, in HPV_DNA_−/p16^INK4a^− OSCC. *P*-values calculated with the log-rank test. *n* denotes the number of patients in each group. HPV_DNA_, human papillomavirus DNA; OSCC, oropharyngeal squamous cell carcinoma.

As described in the introduction, combining HPV_DNA_ and p16^INK4a^ expression as criteria of active HPV infection was proposed. Hence, CD44 intensity staining was examined separately also for patients with HPV_DNA_+/p16^INK4a^+ and patients with HPV_DNA_−/p16^INK4a^− tumors (Fig. 3 and Tables S1 and S2).

Notably, no additional increase in DFS or OS could be observed in the absent/weak CD44 group when subtracting patients with HPV_DNA_+/p16^INK4a^− tumors (Fig. 3A and B) and DFS and OS were similar to that observed for patients with absent/weak intensity staining in Figure 2. However, the cumulative survival rate increased in the medium/strong intensity group in Figure 3A and B, most probably due to the exclusion of all patients with HPV_DNA_+/p16^INK4a^− tumors. Hence, the observed survival difference between absent/weak and medium/strong CD44 intensity staining among HPV_DNA_+/p16^INK4a^+ tumors in Figure 3A and B did not reach statistical significance – although absent/weak CD44 expression in HPV_DNA_+/p16^INK4a^+ OSCC patients showed >95% DFS and OS.

## Discussion

In 290 OSCC patients, we show that those with combined HPV_DNA_+ OSCC and absent/weak CD44 intensity staining presented the best 3-year DFS and OS, with >95% of the patients surviving >3 years after treatment. In addition, HPV_DNA_+ status and absent/weak CD44 intensity staining and p16^INK4a^+ overexpression were also found as independent favorable prognostic markers in OSCC. However, while absent/weak CD44 intensity staining was a positive prognostic marker for both DFS and OS in HPV_DNA_+ OSCC patients, p16^INK4a^ overexpression was only a marker of a favorable OS in these patients.

As mentioned above and shown previously, roughly 80% of all patients with HPV_DNA_+ OSCC may not need the intensified oncological treatment regimes used today 5,6. Nonetheless, additional treatment regimes have already been implemented in many clinics and better stratification markers are therefore needed before a tailored and more deintensified treatment can be introduced. Here, patients with the novel combination of CD44 expression and HPV_DNA_ status showed 95% DFS and 95% OS despite that the majority of the patients were treated only with RT.

In contrast to absent/weak CD44 intensity staining, medium/high CD44 intensity staining correlated to worse prognosis for OSCC in general and for HPV_DNA_+ OSCC. These results are in line with previous reports in HNSCC and other malignancies 9–20. Furthermore, it was proposed that CD44 characterize cancer stem cells in HNSCC 21. In accordance with this, Chen and colleagues demonstrated that CD44-expressing cells displayed cancer stem like properties and had higher RT-resistance in HNSCC 22. Moreover, CD44-expressing cells in HNSCC were demonstrated to have an increased metastatic potential and increased proliferation index 18. Finally, CD44 signaling has also been reported to increase resistance to chemotherapy in HNSCC 23, which together with previously published data and our study all imply CD44 as a negative prognostic factor 8–19.

In our large OSCC cohort, both HPV_DNA_ status and p16^INK4a^ expression correlated independently to a favorable prognosis and overexpression of p16^INK4a^ was significantly correlated to presence of HPV_DNA_, consistent with many previous reports 15–28. Nonetheless, to our knowledge, the significance of p16^INK4a^ overexpression has not been described before separately for HPV_DNA_+ and HPV_DNA_− OSCC. Here, we show that p16^INK4a^ overexpression only influences OS in the HPV_DNA_+ cohort. However, whether the correlation between the absence of p16^INK4a^ expression and a poorer OS in HPV_DNA_+ OSCC is due to the high sensitivity, and to a possibly lower specificity of our HPV_DNA_ detection method, or to actual absence of p16^INK4a^ expression in truly HPV_DNA_+ tumors remains to be elucidated.

In patients, where HPV positive status was defined as HPV_DNA_+ and p16^INK4a^+, CD44 absent/weak staining intensity resulted in a DFS and OS similar to that obtained for patients with HPV_DNA_+ tumors with absent/weak CD44 intensity staining. Furthermore a significant difference between absent/weak and medium high CD44 intensity staining in the HPV_DNA_+ and p16^INK4a^+ patient group was still obtained for DFS, but not for OS. The latter could, however, partly be due to that patients with the poorest outcome (HPV_DNA_+ and p16^INK4a^−) were excluded.

Finally, similar to other studies, a lower age at diagnosis correlated to a favorable DFS and OS, in the whole cohort as well as in patients with HPV_DNA_+ OSCC, while a lower stage correlated to favorable OS in the HPV_DNA_− OSCC patient group 29–30.

Taken together the data suggest that absent/weak CD44 staining in patients with HPV+ OSCC is a strong positive indicator for better clinical outcome irrespective of treatment. Previously, we demonstrated that absent major histocompatibility complex (MHC) class I staining or a high number of CD8 tumor-infiltrating T-lymphocytes were strong prognostic indicators for better clinical outcome in HPV+ OSCC 30–31. It is possible that combining CD44 with these markers may be of even greater benefit for future selection of patients with a favorable outcome.

There are limitations in our study. First, the study was retrospective and the number of patients was limited. Furthermore, treatment was not standardized to a study protocol and the whole patient cohort was not randomized into different treatment arms. Secondly, biopsies were not available for research from all patients; however, we consider the loss of biopsies random as there was, to our knowledge, no systematic loss of specific biopsies. Thirdly, most patients were randomized into two different RT protocols (conventional/hyperfractionated). Nevertheless, improved survival effect was not observed in any of the treatment arms in a study including these patients 32. Finally, here we have only included TSCC and BOTSCC as OSCC and not all other OSCC, but this can also be a benefit as we excluded other OSCC sites where the correlation to HPV and prognosis is more ambiguous 33.

In summary, patients with OSCC with HPV_DNA_+ and absent/weak CD44 intensity staining presented a very high DFS and OS and could potentially be selected as candidates for tailored deintensified treatment. However, our data should be confirmed in a prospective multicentre randomized clinical trial with a larger patient sample, and with other biomarkers, before applied clinically.

## Conflict of Interest

None declared.

## Supporting Information

**Figure S1.** Expression of CD44 and p16^INK4a^ in OSCC. (A–C) show an intense, a weak and an absent CD44 intensity staining, respectively, while (D) shows a positive p16^INK4a^ staining.
