# Supplementary figures and images for: Absent/weak CD44 intensity and positive human papillomavirus (HPV) status in oropharyngeal squamous cell carcinoma indicates a very high survival
Source: Cancer Med. 2013 Jun 14;2(4):507–18. doi: 10.1002/cam4.90 (PMC3799285; doi:10.1002/cam4.90)

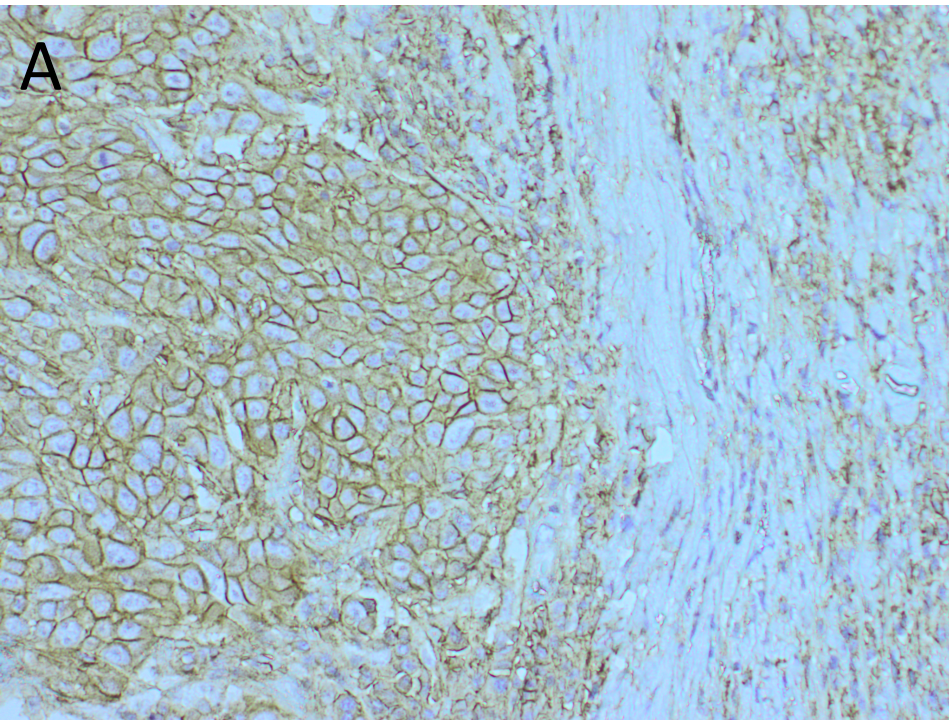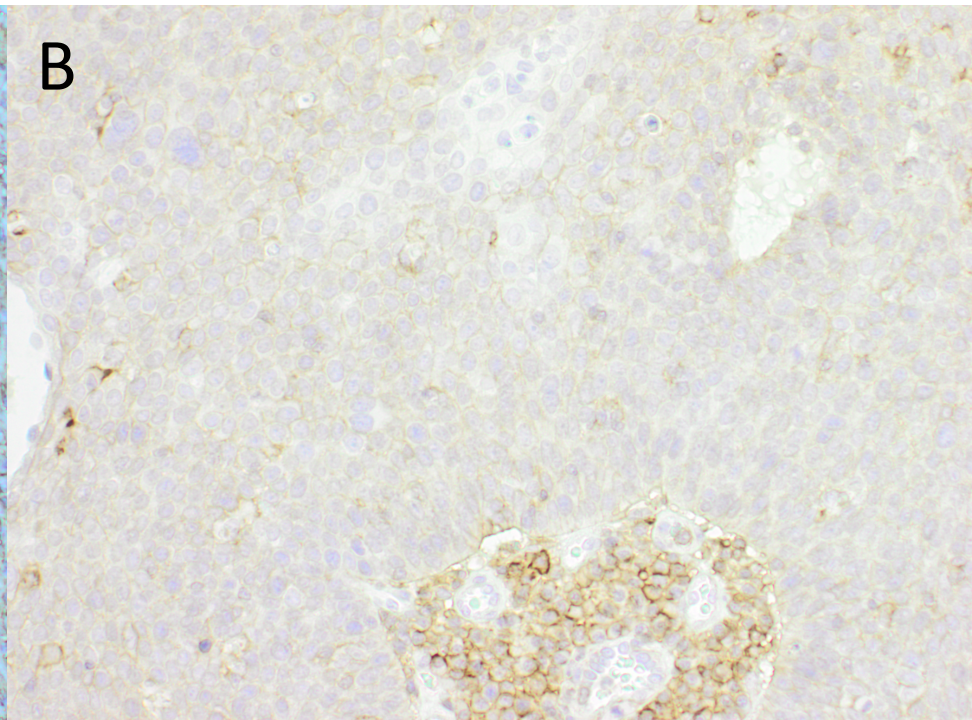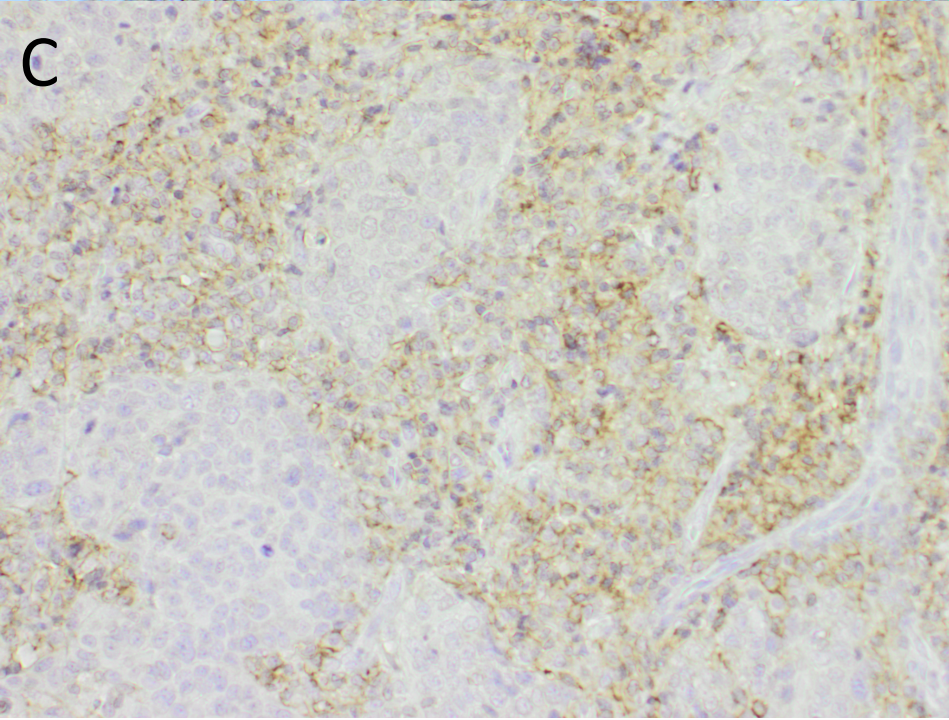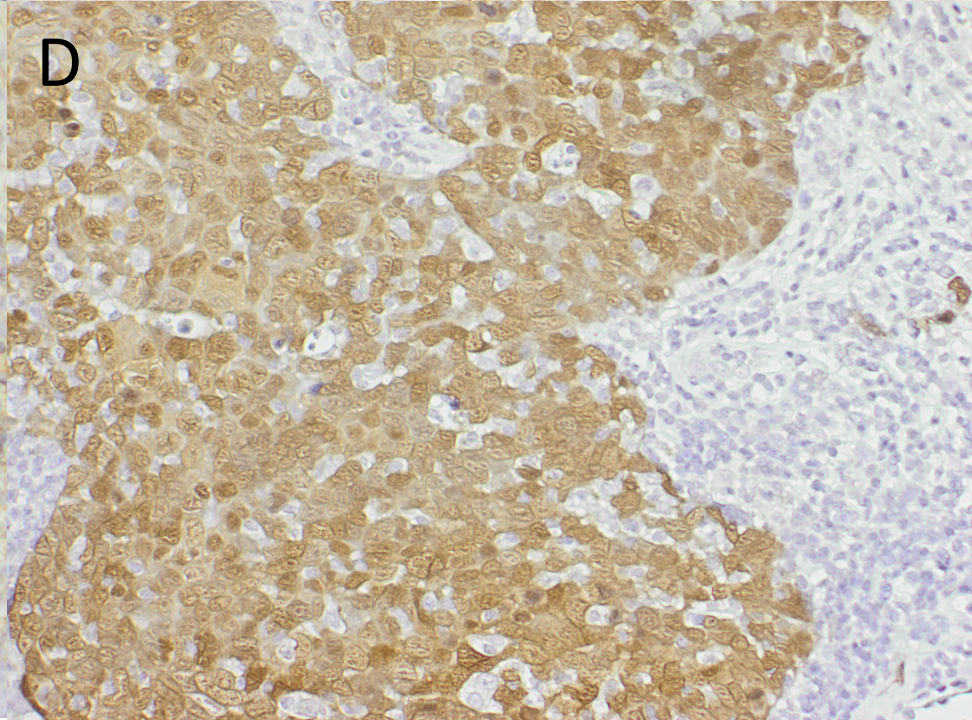

Supplement: Supplementary file 1 — Figure S1. Expression of CD44 and p16INK4a in OSCC. (A–C) show an intense, a weak and an absent CD44 intensity staining, respectively, while (D) shows a positive p16INK4a staining. [file cam40002-0507-SD1.pdf]
